# Association of mast cell density, microvascular density and endothelial area with clinicopathological parameters and prognosis in canine mammary gland carcinomas

**DOI:** 10.1186/s13028-022-00633-2

**Published:** 2022-06-27

**Authors:** Simona Sakalauskaitė, Vita Riškevičienė, Jakov Šengaut, Nomeda Juodžiukynienė

**Affiliations:** 1 Department of Veterinary Pathobiology, Faculty of Veterinary Medicine, Veterinary Academy, Lithuanian University of Health Sciences, Mickevičiaus 9, 44307 Kaunas, Lithuania; 2 Department of Veterinary Medicine, Faculty of Agrotechnologies, University of Applied Sciences, Saltoniškių 58, 08105 Vilnius, Lithuania

**Keywords:** Canine mammary tumours, Endothelial area, Mast cells

## Abstract

**Background:**

Mast cell density has been shown to have both enhancing and inhibiting effects on tumour progression and the ability to predict breast cancer behaviour in humans. However, prognostic results have been contradictory. Some previous studies suggested involvement of mast cells in the progression of canine mammary tumours. This study investigated total, intratumoural and peritumoural mast cell densities by Giemsa staining, and their association with clinicopathological parameters and the disease outcome of canine mammary tumours. In addition, since mast cells promote angiogenesis, the microvascular density and endothelial area were evaluated by CD31 immunostaining.

**Results:**

Intratumoural mast cell density was associated with tumour size, lymph node involvement and tumour-infiltrating lymphocyte count, while peritumoural mast cell density was associated with grade. The endothelial area was associated with grade, mitotic index, tubular formation and proliferation index. Tumours with a high grade, high total intratumoural mast cell density and a larger endothelial area were associated with shorter disease-free survival. Intratumoural mast cell density and grade were found to be independent prognostic factors.

**Conclusions:**

These results suggest that intratumoural mast cell density and the endothelial area can be used to evaluate the aggressiveness of canine mammary carcinomas, while intratumoural mast cell density could be of use as an independent predictor of a prognosis of disease-free survival. Peritumoural mast cell density does not seem to influence tumour behaviour.

## Background

Canine mammary gland tumours (CMTs) are one of the most frequently diagnosed neoplasms in female dogs [1]. Multiple parameters, such as histological type, grade, stage, lymph node involvement and proliferation index (PI), are considered to be prognostic factors [2–5]. Problems arise because CMTs exhibit diverse biological behaviour, while some parameters, such as type and grade, are prone to the observer’s subjectivity, thus resulting in the need for more objective prognostic factors [6, 7]. Mast cell density (MCD) has been proposed as a prognostic factor since it is a widely researched parameter in various human cancers, including breast cancer (BC). However, the effect of mast cells (MCs) on tumour progression is contradictory and seemingly dependent on the cancer type [8–10]. Moreover, the effect of MCs on cancer progression may depend on their location in the tumour. Peritumoural mast cells have been found to exhibit a cytolytic effect on breast tumour cells [11]. However, another study found them to promote metastatic spread, while intratumoural MCs enhance lymphatic invasion [12]. MCs release a variety of mediators that have either tumour growth-promoting capabilities or anti-cancer effects [13]. For example, these cells release neutral proteases that contribute to the dissolution of the extracellular matrix and basal membrane components, allowing for tumour invasion and metastasis [14–16]. Furthermore, MCs are associated with tumour angiogenesis in BC and CMTs, which is known to play an important role in tumour progression [17, 18].

Angiogenesis, measured as microvascular density (MVD), has been found to be significantly higher in malignant mammary tumours with lymph node metastasis than in benign tumours and is considered to be a significant prognostic factor in benign and malignant CMTs [19–21]. Furthermore, angiogenesis can be measured as an endothelial area (EA) to reduce the subjectivity that could arise from counting single vessels in an area of tangled vessels [22]. EA has been found to be an independent prognostic factor for invasive BC and is hypothesised to be a better prognostic factor in human renal cell carcinoma than MVD [22, 23]. In dogs, MVD and EA have been found to correlate in non-Hodgkin’s lymphoma, with both parameters increasing with a higher malignancy grade [24], although in CMTs the endothelial area was not found to be a significant prognostic factor of overall survival (OS) [19]. Despite the evidence of the importance of mast cells and angiogenesis in cancer development, to the authors’ knowledge there have been just a few studies investigating MCs in CMTs. Tryptase-positive MCs were found to be increased in the peritumoural stroma of CMTs with the highest number at the invasive margin, but EPOR (erythropoietin receptor)-positive MCs count was significantly decreased in the tumours [25]. It has been shown that there is a positive correlation between MCs and MVD [26], and a significantly higher correlation was found between tryptase-positive MCs and MVD [18]. Also, it was shown that MCs density varies depending on the malignancy grade of CMTs [27], however in another study MCs were not associated neither with more or less aggressive canine mammary neoplasms [28]. Only one study assessing MCs was prognostic and showed that low stromal MC density predicted a poor outcome in malignant CMTs [8]. Furthermore, angiogenesis measured as EA has been investigated in only two studies, with one of them being prognostic [19, 29].

Therefore, the aim of the present study was to investigate total (TMCD), intratumoural (IMCD) and peritumoural (PMCD) mast cell density and angiogenesis, measured as microvascular density and the endothelial area, in canine mammary carcinomas (CMCs) and their association with clinicopathological parameters, and to evaluate TMCD, IMCD, PMCD, MVD and EA as potential prognostic factors.

## Methods

### Sample collection

A total of 134 CMCs were collected for this study from the archives of the Pathology Centre, Veterinary Academy, Lithuanian University of Health Sciences. All the tumours had been submitted for routine histopathology after surgical tumour excision as part of a treatment plan. No tumours were excised solely for research or diagnostic purposes and no samples were specifically collected for this study, therefore ethical approval was waived. Information about the dogs’ age and breed was collected from the archive of the Pathology Centre or from the referring veterinarian. Additional information about the tumour size and lymph nodes being positive for metastasis or negative with no metastatic infiltration was available in 45 samples. All the samples were fixed in 10% buffered formalin, embedded in paraffin blocks, cut into 6 µm sections and placed on silane-coated slides for histopathology, immunohistochemistry for detection of the proliferation index (PI) by Ki67, microvascular density (MVD) and endothelial area (EA) by CD31 and histochemistry for mast cell (MC) count.

### Histopathological examination

**Fig. 1:**
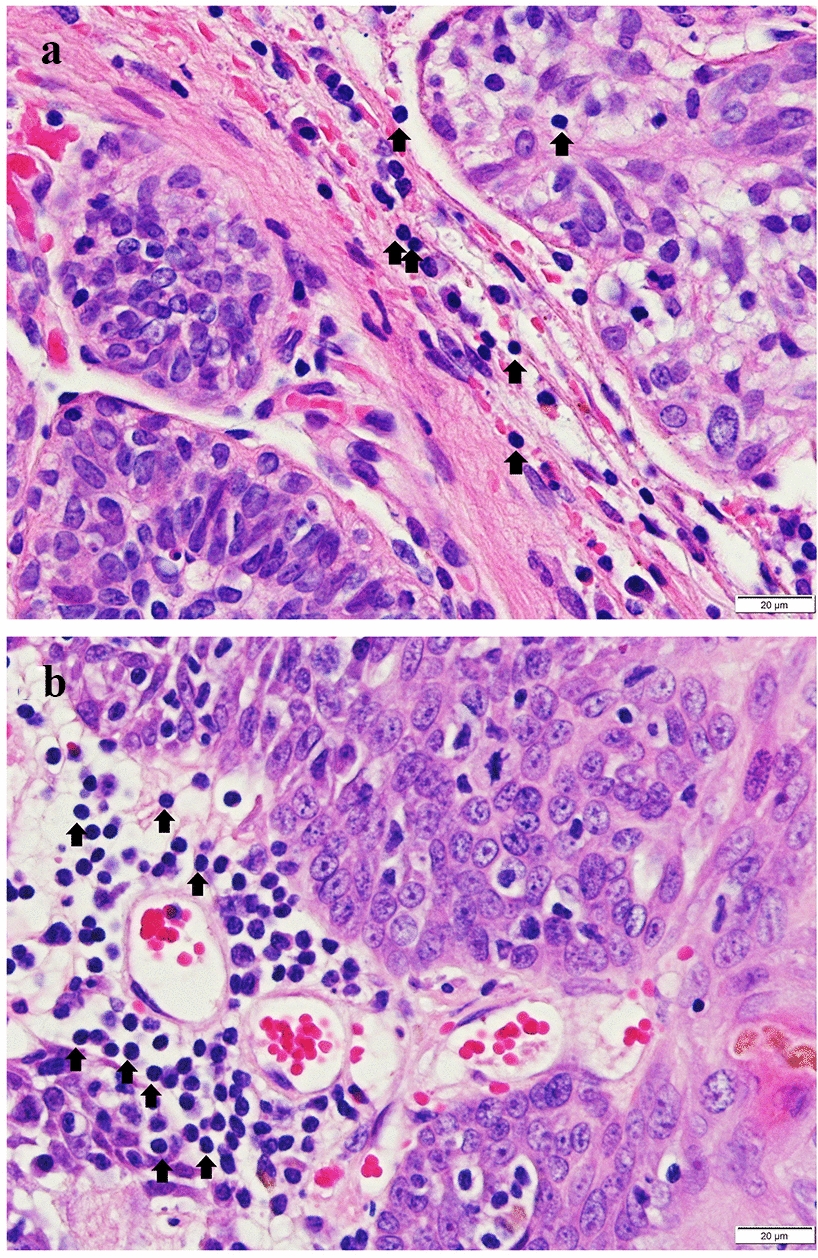
Tumour-infiltrating lymphocytes (TILs) (arrows) in canine mammary carcinomas. **a** Carcinoma with average count of TILs. **b** Carcinoma with high count of TILs. Bar, 20 µm

The slides for initial classification were stained with hematoxylin and eosin (H&E). Histologically the tumours were classified according to Goldschmidt et al. [30]. The grading was undertaken in accordance with the modified Elston and Ellis method [31] when evaluating tubule formation, nuclear pleomorphism and mitotic index. The other clinicopathological parameters evaluated were the presence of necrosis and the tumour-infiltrating lymphocyte (TIL) count. TILs included lymphocytes and plasma cells, but excluded macrophages. Tumour-infiltrating lymphocytes were counted in three hotspots (areas highest in TILs count) at × 400 magnification (high-power field [HPF]). According to cut-off points, tumours with ≤ 20 cells/HPF were considered low in TILs, ≤ 50 cells/HPF were categorised as average and > 50 cells/HPF were categorised as high (Fig. 1). The cut-off values were derived from the tertile boundaries in the results.

### Immunohistochemistry

Immunohistochemistry for Ki67 and CD31 was applied for all the samples. A negative control was obtained by omitting the primary antibody, while cutaneous tumours were used as a positive control for Ki67 and hemangiomas were used as control for CD31 detection. The tumour slides were deparaffinised in xylene, rehydrated with degraded ethanol, and washed with running and distilled water. Antigen retrieval was performed by immersing the slides in the Target Retrieval Solution (Dako Denmark A/S, Glostrup, Denmark) with pH 6.0 for Ki67 and pH 9.0 for CD31 at 96 °C for 40 min. The EnVision FLEX+, High pH (Link) (Dako Denmark A/S, Glostrup, Denmark) visualisation system was used. Washing between every step was undertaken with a wash buffer. Endogenous peroxidase was blocked by incubation of sections in a peroxidase blocking reagent for 10 min at room temperature (RT). The samples were incubated for 30 min at RT with primary monoclonal antibodies: Ki67 (clone MIB-1, Dako, Glostrup, Denmark; dilution: 1:150) and CD31 (clone JC70A, Dako, Glostrup, Denmark; dilution 1:40). Samples were subsequently incubated with EnVision FLEX + Mouse (Linker) and secondary antibody (EnVision/HRP) for 30 min at RT. The colour visualisation was reached with 3,3-diaminobenzidine tetrahydrochloride (DAB + Chromogen). Sections were counterstained with Mayer’s Hematoxylin Solution (Sigma-Aldrich, Saint Louis, MO, USA), dehydrated with xylene, cover-slipped and evaluated by light microscopy (Olympus BX36, Tokyo, Japan).

### Image analysis

The measurement of microvascular density (MVD) by CD31 was performed at ten representative microscopical fields, according to Weidner’s method [32]. All positive endothelial cells or cell clusters clearly separated from adjacent microvessels, tumour cells and other connective-tissue elements were considered as a single microvessel. Each slide was scanned at low magnification to identify hotspots (areas with the highest number of vessels). Digital images of these hotspots were taken at × 400 magnification (HPF) and the number of microvessels counted per 1 mm^2^.

The endothelial area (EA) in samples immunostained with CD31 was also evaluated using ImageJ software (National Institutes of Health, Bethesda, MD, USA and Laboratory for Optical and Computational Instrumentation, University of Wisconsin, Madison, WI, USA). The perimeter of each single vessel was delimitated and the corresponding area was measured until the area of all vessels observed in the 1 mm^2^ sample was calculated. Vessels in necrotic areas were not counted.

**Fig. 2:**
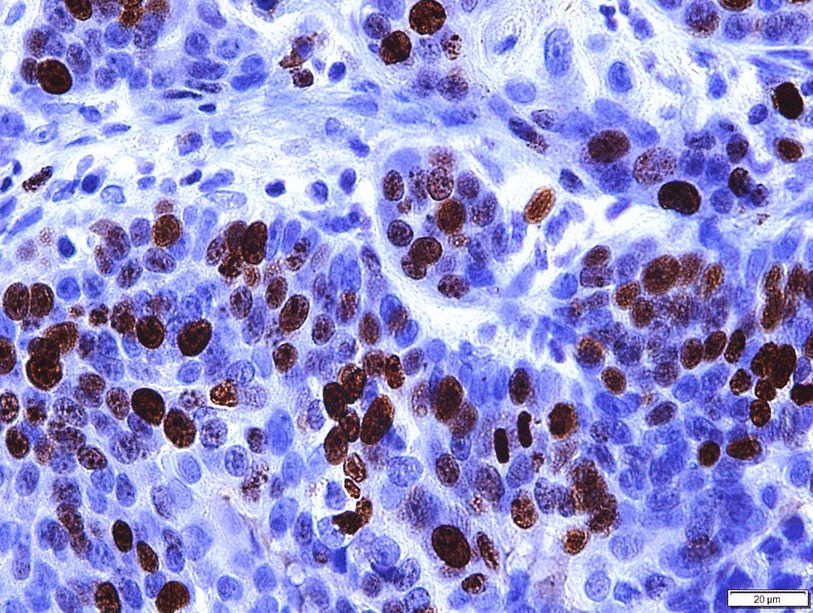
Immunohistochemical reaction for Ki67 (brown) using MIB-1 antibody in a canine mammary carcinoma. Bar 20 µm

Evaluation of Ki67 was done according to recommendations from the International Ki67 in Breast Cancer working group [33]. The evaluation was undertaken by counting 1000 cells in sample areas representing the hotspots (areas with the highest immunoreactivity for Ki67). Immunoreactivity was considered when cells had a stained nucleus, regardless of its staining intensity (Fig. 2). The Ki67 score (PI) was expressed as the percentage of positively stained cells out of the total 1000 tumour cells counted.

### Mast cell staining and quantification

Staining for mast cells (MCs) was performed with Giemsa Stain Modified Solution (Sigma-Aldrich, Egham, Surrey, UK). The slides were deparaffinised in xylene and isopropanol, rehydrated with 96% ethanol and washed in running tap water. The slides were subsequently left in Giemsa stain for 1 h. They were then submerged for 10–12 s in acidic water and 96% ethanol for colour differentiation and completion. Samples were cover-slipped and scanned at × 100 magnification for areas of the greatest abundance of MCs in the intratumoural and peritumoural zones. The intratumoural zone was defined according to Glajcar et al. [34]. Mast cells located within the centre of neoplastic tissue and more than 1 HPF (0.2 mm^2^ field area) inside the tumour edge were considered as the intratumoural population, while MCs no further than (1) HPF from the tumour margins and no more than (2) HPF outside the tumour margins were considered to reside in the peritumoural zone. Total mast cell density was counted as the sum of MCs in the intratumoural and peritumoural zones. MCs were counted in non-overlapping HPFs in a 1 mm^2^ sample.

### Follow-up

With the consent of the owners and referred veterinarians, 35 dogs were included in the follow-up. This was performed according to the clinics’ practice and no dog received additional evaluation or treatment for cancer and metastasis than is routinely the case, therefore ethical approval was waived. Physical examinations, thoracic radiographs and abdominal ultrasounds were undertaken for evaluation of distant metastasis every 6 months, or when owners raised concerns, during a 2-year period. In the event of enlarged lymph nodes, an evaluation was undertaken by cytology after fine-needle aspiration and/or resection of the lymph nodes for histopathology assessment if a second surgery was needed due to a recurrence. Disease-free survival (DFS) was calculated from the date of surgery to the date of detection of the first local recurrence or distant metastasis. Patients were censored if they were free of recurrence/metastasis at the end of 2-year period. No dogs were lost to follow-up or died due to a cause unrelated to the tumour. Only one dog was euthanised due to tumour metastasis, therefore overall survival (OS) was not counted.

### Statistical analysis

Non-normally distributed data (TMCD, PMCD, SMCD) are expressed as the median (interquartile range [IQR]). Normally distributed data (MVD, EA, PI) are expressed as the mean ± standard deviation (SD). The associations between total (TMCD), peritumoural (PMCD) and intratumoural mast cell (IMCD) densities and clinicopathological parameters were assessed using the Kruskal–Wallis H test with post hoc analysis by the Mann–Whitney test. The associations between microvascular density (MVD), endothelial area (EA) and clinicopathological parameters were assessed by ANOVA with Bonferroni post hoc analysis. The correlations between MC densities, MVD and EA were analysed by Pearson’s correlation. The difference between IMCD and PMCD was analysed by Wilcoxon signed-rank test. Survival curves were calculated by the Kaplan–Meier method using mean values as the cut-off, and the log-rank test was used to analyse differences between the groups defined for each variable. The risk of local recurrence/metastasis was estimated by Cox regression analysis. Multivariable Cox regression analysis was also applied, including factors significant in the univariate analysis, using a Forward Stepwise (Likelihood Ratio) method. The significance level was set at P < 0.05. Statistical analysis was performed using SPSS software version 25.0 (IBM, Chicago, IL, USA).

## Results

### Clinicopathological characteristics of tumours

**Fig. 3:**
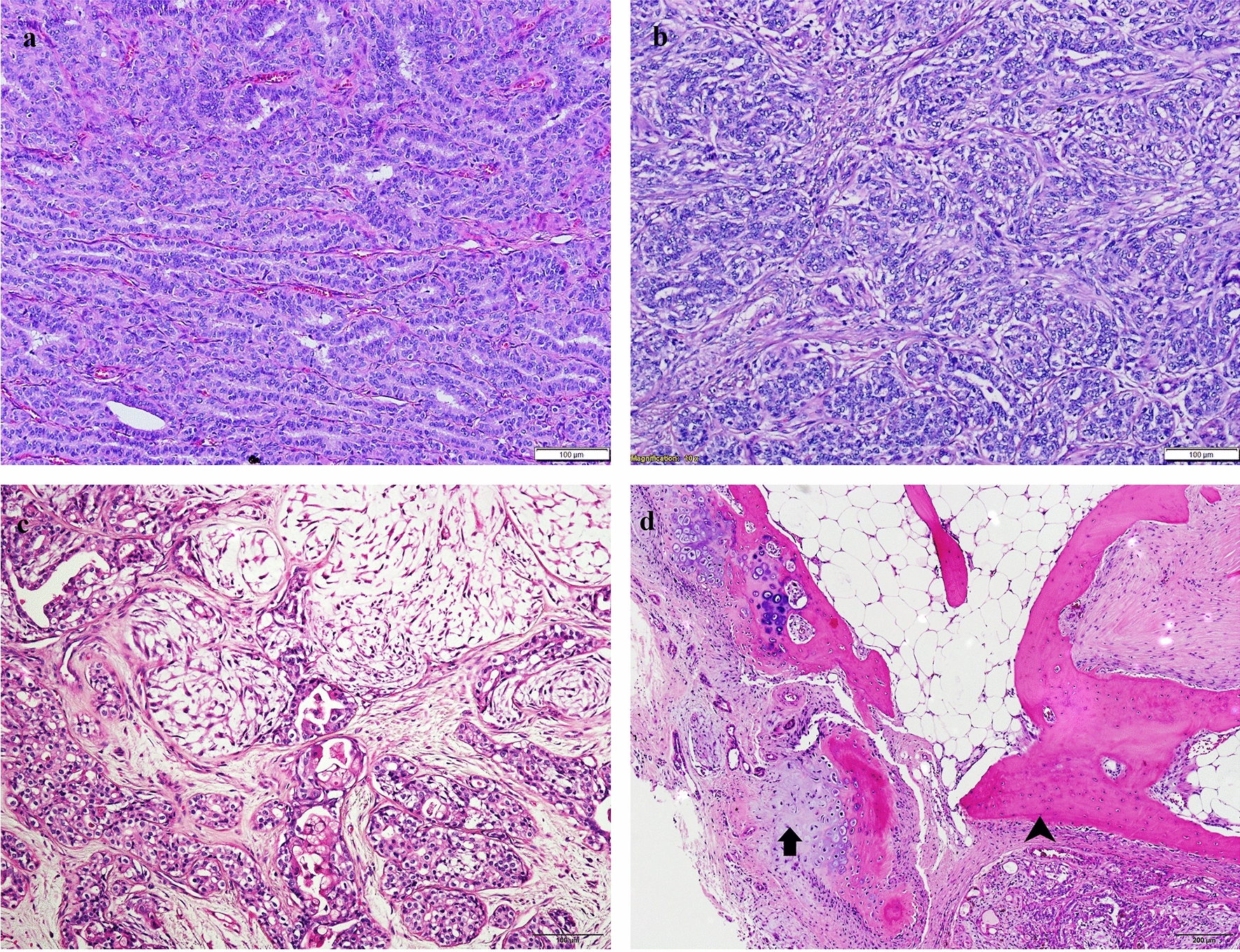
The most common histological types of canine mammary carcinomas in our study. **a** Simple; bar 100 µm. **b** Solid; bar 100 µm. **c** Complex; bar 100 µm. **d** Mixed with cartilage tissue (arrow) and osseous tissue (arrowhead); bar 200 µm

A total of 134 canine mammary carcinomas (CMCs) were investigated. The age of the dogs ranged from 3 to 16 years, with an average of 9.72 ± 2.68 years. The tumours included in this study were diagnosed as follows: simple carcinomas (n = 24), solid carcinomas (n = 19), comedocarcinomas (n = 9), carcinomas arising in a complex/mixed tumour (n = 3), complex carcinomas (n = 30), mixed carcinomas (n = 32), intraductal papillary carcinomas (n = 13) and adenosquamous carcinomas (n = 4). Examples of most commonly diagnosed histological types can be seen in Fig. 3. The grade of the CMCs was determined: I (n = 74), II (n = 35) and III (n = 27). The average proliferation index (PI) was 14.68 ± 12.85%. Grade III carcinomas had a higher PI (23.12 ± 11.72) than grade I (10.9 ± 11.47) or grade II (16.34 ± 13.39) carcinomas (P < 0.0001). Also, tumours with necrosis had higher PI (20.7 ± 12.85) than neoplasms without necrosis (9.74 ± 13.95) (P < 0.0001). PI was associated with mitotic index (P < 0.0001): as mitotic index increased proliferation index also became higher.

### Distribution of mast cells and their association with clinicopathological features

**Fig. 4:**
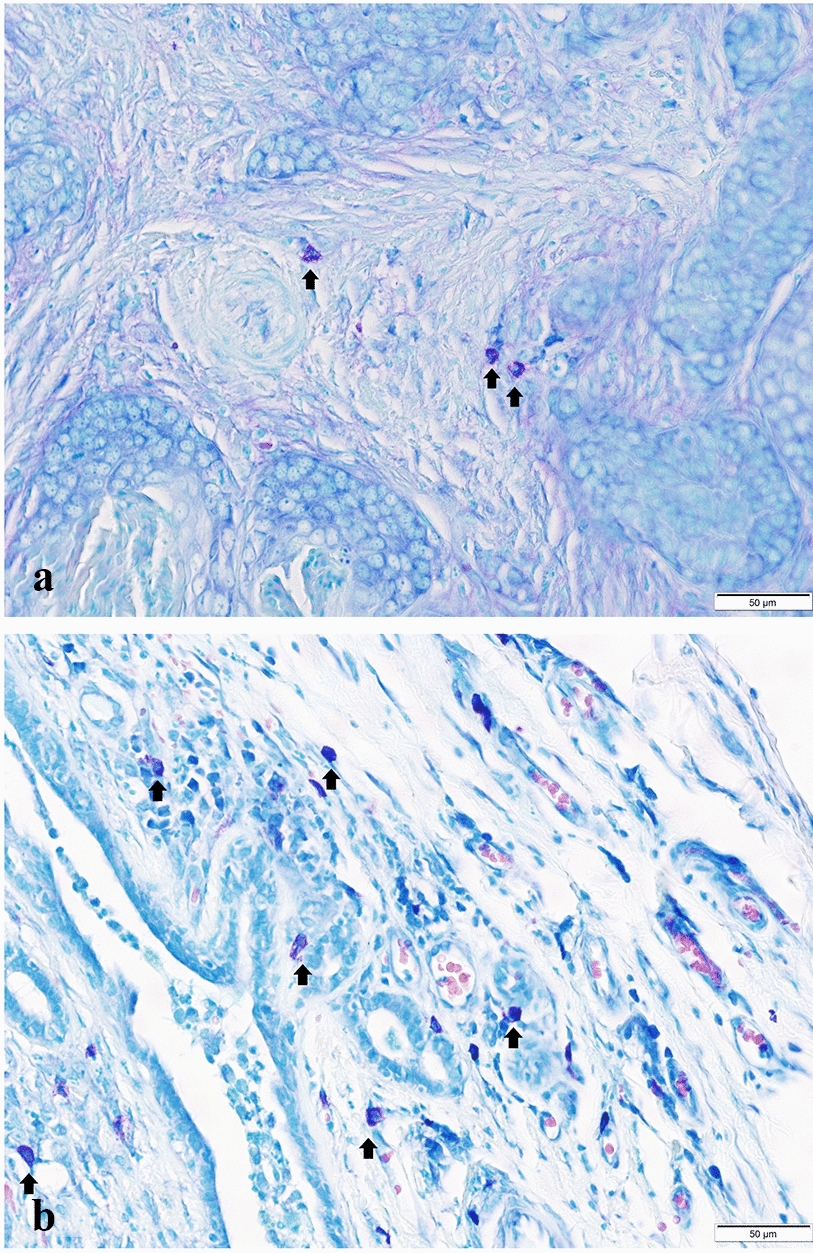
Mast cells (arrows) in canine mammary carcinoma. **a** Intratumoural. **b** Peritumoural. Bar 50 µm

The median TMCD in the carcinomas was 168 (147)/1 mm^2^. Mast cells were mostly distributed in the periphery of the tumours and rarely in the intratumoural stroma (Fig. 4). PMCD [136 (115)/1 mm^2^] was higher (P < 0.0001) than IMCD [41 (62)/1 mm^2^]. The highest TMCD was in the simple carcinoma, however there were no significant differences between any MC density in different histological types of CMTs (Table 1). We did not find correlation between any MC density and PI. There were no significant associations between PMCD and any parameters observed except grade, while IMCD was higher in larger tumours (P = 0.02), in tumours with lymph nodes metastasis (P = 0.024) and in tumours with high TIL infiltration (P = 0.41). All associations are detailed in Table 1.

**Table 1:** Associations between mast cell density and clinicopathological parameters in canine mammary carcinomas

Clinicopathological parameters	Total mast cells [median (IQR)/1 mm^2^]	P	Peritumoural mast cells [median (IQR)/1 mm^2^]	P	Intratumoural mast cells [median (IQR)/1 mm^2^]	P
Histological type (n = 134)						
Carcinoma simple (n = 24)	225 (132)	NS	180 (181)	NS	60 (64)	NS
Carcinoma solid (n = 19)	168 (160)		144 (124)		50 (66)	
Comedocarcinoma (n = 9)	138 (234)		120 (165)		18 (71)	
Carcinoma arising in a complex/mixed tumour (n = 3)	122^1^		98^1^		29^1^	
Carcinoma complex (n = 30)	138 (108)		113 (100)		31.5 (43)	
Carcinoma mixed (n = 32)	152 (187)		127 (165)		18.5 (60)	
Intraductal papillary carcinoma (n = 13)	106 (189)		100 (105)		22 (47)	
Adenosquamous carcinoma (n = 4)	195 (108)		147 (85)		48 (58)	
Histological grade (n = 134)						
Grade I (n = 73)	150 (153)	NS	114 (122)^a^	0.041	29 (62)	NS
Grade II (n = 34)	173 (194)		164 (170)^a^		50 (53)	
Grade III (n = 27)	168 (170)		140 (116)		48 (75)	
Tumour necrosis (n = 134)						
Absent (n = 73)	164 (161)	NS	136 (119)	NS	35 (65)	NS
Present (n = 61)	168 (167)		136 (119)		48 (59)	
Tumour-infiltrating lymphocytes (n = 112) lymphocytes (n = 114)						
Low (n = 55)	140 (142)	NS	100 (114)	NS	30 (49)^a^	0.41
Moderate (n = 38)	172 (142)		137 (128)		28.5 (42)	
High (n = 19)	194 (118)		153 (106)		64 (74)^a^	
Proliferation index (n = 134)						
≤ 11% (n = 66)	144 (156)	NS	118.5 (138)	NS	26 (63)	NS
> 11% (n = 67)	170 (134)		142 (84)		50 (61)	
Tumour size (n = 45)						
T1 < 3 cm (n = 11)	90 (153)	NS	76 (155)	NS	18 (46)	0.02
T2 3–5 cm (n = 10)	152 (156)		127.5 (142)		15 (40)^a^	
T3 > 5 cm (n = 24)	174 (158)		118 (120)		50 (66)^a^	
Lymph node metastasis (n = 45)						
No (n = 35)	111 (155)	NS	97.5 (144)	NS	14 (19)	0.024
Yes (n = 10)	166 (127)		118 (118)		48 (55)	

Superscript letter denotes statistically significant differences between exact subgroups

*P* statistical significance, *NS* not significant

^1^Interquartile range is not calculated due to the small sample size in the subgroups

### Endothelial area and microvascular density associations with clinicopathological parameters

**Fig. 5:**
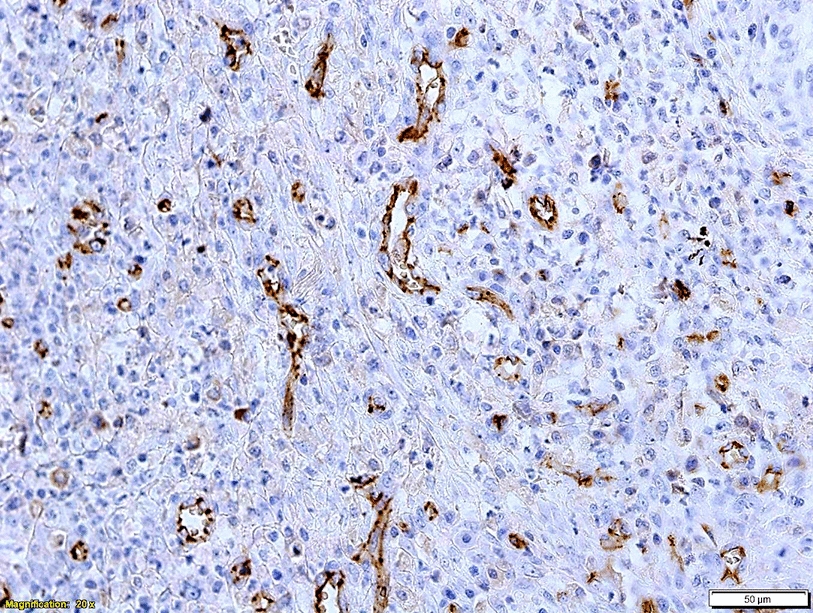
Microvessels in canine mammary carcinoma identified by immunohistochemical labelling of endothelial cells for CD31 expression (brown). Bar 50 µm

CD31-positive endothelial cells were labelled brown (Fig. 5). The mean microvascular density (MVD) was 86.99 ± 43.29/1 mm^2^. The mean endothelial area (EA) was 2449.14 × 10^–2^ ± 575.39 µm/mm^2^. A positive correlation was found between MVD and EA (r = 0.414, P < 0.001). Furthermore, there was a positive correlation between mean MVD and TMCD (r = 0.226; P = 0.009) and PMCD count, (r = 0.177; P = 0.041) but not with IMCD. There was no significant correlation between any type of MC density and EA. Tumours with a higher EA tended to have associations with parameters (high grade, mitotic index, PI and decreased tubular formation) characteristic of more aggressive tumours, while MVD was only associated with the presence of necrosis (P = 0.047). All the observed associations are described in Table 2.

**Table 2:** Associations between endothelial area, microvascular density and clinicopathological parameters in canine mammary carcinomas

Clinicopathological parameters	Endothelial area (mean ± SD/1 mm^2^)	P	Microvascular density (mean ± SD/1 mm^2^)	P
Histological type (n = 134)				
Carcinoma simple (n = 24)	2370.59 × 10^–2^ ± 546.39 µm	NS	115.68 ± 51.74	NS
Carcinoma solid (n = 19)	2632.44 × 10^–2^ ± 519.34 µm		81.94 ± 32.23	
Comedocarcinoma (n = 9)	2787.11 × 10^–2^ ± 829.01 µm		89.79 ± 37.99	
Carcinoma arising in a complex/mixed tumour (n = 3)	2549.33 × 10^–2^ ± 564.63 µm		51.32 ± 3.51	
Carcinoma complex (n = 30)	2420.02 × 10^–2^ ± 569.64 µm		77.05 ± 32.69	
Carcinoma mixed (n = 32)	2189.73 × 10^–2^ ± 438.96 µm		82.11 ± 37.58	
Intraductal papillary carcinoma (n = 13)	2574.37 × 10^–2^ ± 492.51 µm		73.04 ± 30.42	
Adenosquamous carcinoma (n = 4)	3100.95 × 10^–2^ ± 804.37 µm		118.25 ± 111.5	
Histological grade (n = 13)				
Grade I (n = 73)	2283.02 × 10^–2^ ± 516.64 µm^a^	0.001	90.4 ± 44.12	NS
Grade II (n = 34)	2511.01 × 10^–2^ ± 526.49 µm		87.72 ± 48.73	
Grade III (n = 27)	2820.39 × 10^–2^ ± 613.56 µm^a^		78.86 ± 32.3	
Tumour necrosis (n = 134)				
Absent (n = 73)	2401.42 × 10^–2^ ± 581.02 µm	NS	80.96 ± 39.28	0.047
Present (n = 61)	2506.26 × 10^–2^ ± 567.85 µm		94.22 ± 46.96	
Tumour-infiltrating lymphocytes (n = 112)				
Low (n = 55)	2447.44 × 10^–2^ ± 630.6 µm	NS	89.8 ± 46.99	NS
Moderate (n = 38)	2441.75 × 10^–2^ ± 556.49 µm		90.01 ± 39.22	
High (n = 19)	2321 × 10^–2^ ± 484.13 µm		86.11 ± 50.37	
Mitotic index (n = 134)				
Low (n = 67)	2278.36 × 10^–2^ ± 534.91 µm^a^	0.001	90.94 ± 49.05	NS
Moderate (n = 27)	2532.93 × 10^–2^ ± 465.75 µm		89.1 ± 41.01	
High (n = 40)	2678.64 × 10^–2^ ± 625.2 µm^a^		78.96 ± 33.21	
Nuclear pleomorphism (n = 134)				
Mild (n = 16)	2318.88 × 10^–2^ ± 483.38 µm	NS	85.34 ± 31.25	NS
Moderate (n = 82)	2397.22 × 10^–2^ ± 577.76 µm		57.74 ± 42.91	
Marked (n = 36)	2625.31 × 10^–2^ ± 582.27 µm		86.02 ± 49.4	
Tubular formation (n = 134)				
Marked (n = 79)	2302.75 × 10^–2^ ± 519.61 µm^a^	0.003	88.07 ± 44.42	NS
Moderate (n = 20)	2507.79 × 10^–2^ ± 513.41 µm		87.88 ± 32.17	
Mild (n = 35)	2746.06 × 10^–2^ ± 574.39 µm^a^		86.89 ± 43.28	
Proliferation index (n = 134)				
≤ 11% (n = 66)	2342.96 × 10^–2^ ± 576.48 µm	0.031	85.9 ± 48.4	NS
> 11% (n = 67)	2557.68 × 10^–2^ ± 561.58 µm		88.74 ± 37.88	
Tumour size (n = 45)				
T1 < 3 cm (n = 11)	2129.6 × 10^–2^ ± 412.69 µm	NS	85.53 ± 62.2	NS
T2 3–5 cm (n = 10)	2611.14 × 10^–2^ ± 443.57 µm		84.08 ± 32.58	
T3 > 5 cm (n = 24)	2472.56 × 10^–2^ ± 586.65 µm		90.53 ± 54.98	
Lymph node metastasis (n = 45)				
Yes (n = 10)	2440.42 × 10^–2^ ± 543.54 µm	NS	89 ± 51.06	NS
No (n = 35)	2374.24 × 10^–2^ ± 543.52 µm		82.13 ± 55.35	

Superscript letter denotes statistically significant differences between exact subgroups

*P* statistical significance, *NS* not significant

### Disease-free survival analysis

In this study, 15/35 (45%) of dogs included in the follow-up developed a recurrence or distant metastasis, however only one dog was euthanised due to pulmonary metastasis during the follow-up period. The mean disease-free survival (DFS) time was 19.9 ± 8.26 months. Within the dog group that developed recurrence or distant metastasis, 4 out of 15 (26.7%) patients had been spayed.

The histological types of the carcinomas included in the follow-up were simple (n = 7), solid (n = 11), intraductal-papillary (n = 3), complex (n = 8) and mixed (n = 6). No observed clinicopathological parameters (histological type, tumour size, lymph node metastasis, PI, presence of necrosis, inflammation or TILs) were associated with DFS time except histological grade (log rank; P = 0.043). Grade III tumours had a shorter mean DFS time and a 6.46 times greater risk of local recurrence/distant metastasis (P = 0.024) than grade I tumours (Table 3).

**Table 3:** Associations of grade, mast cell density, microvascular density and endothelial area with disease-free survival

Covariate	Recurrence or distant metastasis rate	Mean DFS time in months (95% CI)	p^a^	Univariate	p	Multivariable^b^	p
				Hazard ratio (95% CI)		Hazard ratio (95% CI)	
Grade							
I	2/10 (20%)	23.9 (23.8–24)		Referent		Referent	
II	7/15 (46.7%)	20 (17.1–22.9)		3.64 (0.75–17.71)	NS	2.72 (0.55–15.54)	NS
III	6/10 (60%)	14 (8.1–19.9)	0.043	6.46 (1.29–32.49)	0.024	7.04 (1.34–36.92)	0.021
TMCD (mm^2^)							
Low (< 154.4 cells)	4/18 (32.2%)	20.7 (17.6–23.8)		Referent			
High (> 154.4 cells)	11/17 (64.7%)	18.3 (14.7–22)	0.032	3.25 (1.03–10.25)	0.044		NS
IMCD (mm^2^)							
Low (< 48.8 cells)	6/23 (26.1%)	20.5 (17.5–23.4)		Referent		Referent	
High (> 48.8 cells)	9/12 (75%)	17.7 (13.7–21.8)	0.008	3.78 (1.32–10.84)	0.014	4.1 (1.31–12.75)	0.015
PMCD (mm^2^)							
Low (< 110.7 cells)	6/19 (31.6%)	21.6 (19.5–23.8)					
High (> 110.7 cells)	9/16 (56.2%)	17 (12.7–21.3)	NS		NS		NS
MVD (mm^2^)							
Low (< 98.4 vessels)	7/19 (37.8%)	20.6 (17.8–23.3)					
High (> 98.4 vessels)	8/16 (50%)	18.3 (14.2–22.4)	NS		NS		NS
EA (mm^2^)							
Low (< 2437.9 × 10^–2^)	7/21 (33.3%)	21.9 (19.8–24)					
High (> 2437.9 × 10^–2^)	8/14 (61.1%)	16 (11.4–20.6)	0.044		NS		NS

*P* statistical significance, *NS* not significant, *CI* confidence interval, *DFS* disease-free survival

^a^Log rank test

^b^Multivariable Cox Proportional Hazard Model with Forward Stepwise Method (Likelihood ratio)

**Fig. 6:**
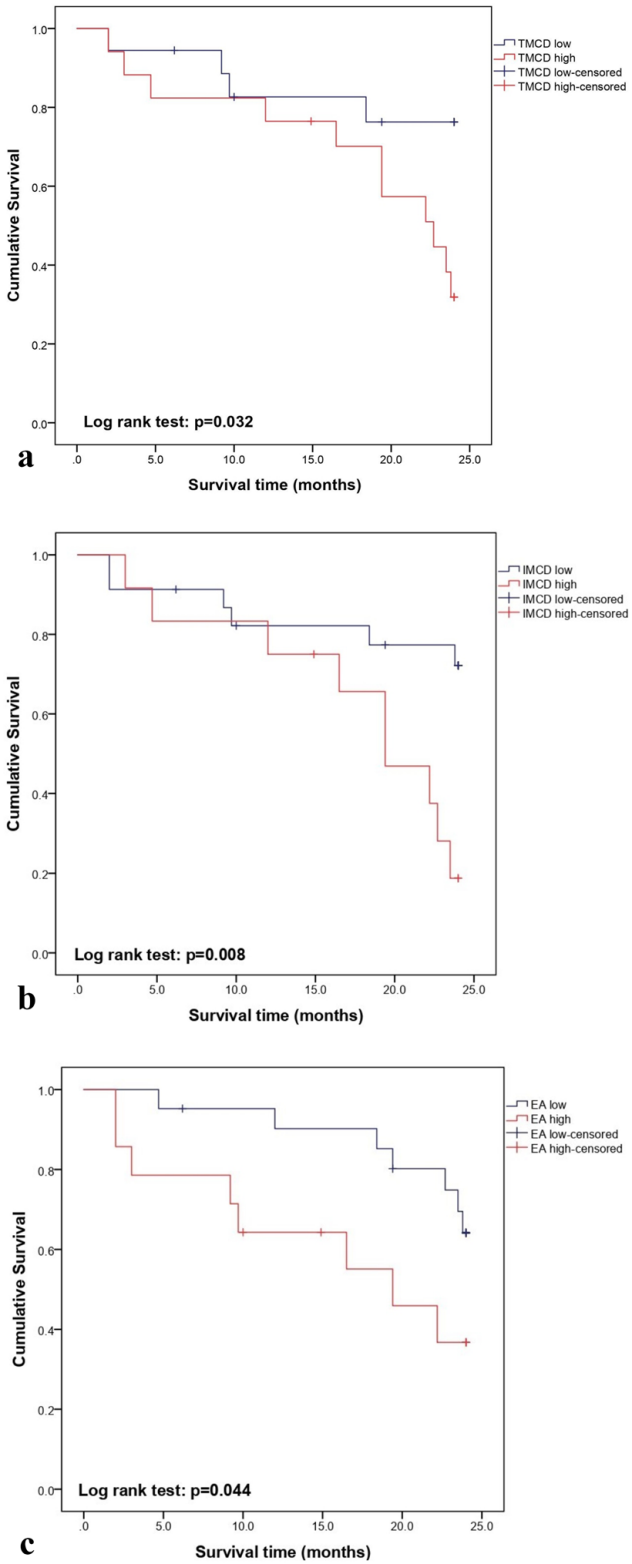
Kaplan–Meier disease-free survival curves comparing canine mammary carcinomas with: **a** low and high total mast cell density. TMCD low, total mast cell density low (< 154.4 cells/mm^2^); TMCD high, total mast cell density high (> 154.4 cells/mm^2^). **b** Low and high intratumoural mast cell density. IMCD low, intratumoural mast cell density low (< 48.8 cells/mm^2^); IMCD high, total mast cell density high (> 48.8 cells/mm^2^). **c** Low and high endothelial area. EA low, endothelial area low (< 2437.9 × 10^–2^ mm^2^); EA high, endothelial area high (> 437.9 × 10^–2^ mm.^2^)

Tumours with a high TMCD (log rank; P = 0.032; Fig. 6a) and IMCD (log rank; P = 0.08; Fig. 6b) had a shorter DFS time. PMCD was not significantly associated with DFS time. Furthermore, tumours with a high EA (log rank; P = 0.044; Fig. 6c) were associated with a shorter DFS time, in contrast to MVD (Table 3). However, the increased risk of recurrence/distant metastasis was only associated with TMCD (P = 0.044) and IMCD (P = 0.014) count. Multivariable analysis showed that only a high IMCD and grade III retained statistical significance as independent predictors of a shorter DFS (Table 3).

## Discussion

Mast cells produce many different mediators (vascular endothelial growth factor (VEGF), tumour necrosis factor, interleukin-6, tryptase, histamine etc.) that can modify the tumour microenvironment, for example by promoting vascular development [35, 36]. MCs are widely described in different studies of various types of malignant tumours in humans, including BC, however the pro-tumourigenic and anti-tumourigenic effects of MCs are contradictory and seem to be affected by MC localisation [10]. The present study found that IMCD was significantly lower than PMCD, which is in agreement with a previous study that compared tryptase-positive MC density in intratumoural and peritumoural zones in different types of canine mammary carcinomas [27]. In a previous study [18] none of the MC densities investigated had a significant association with histological type. Interestingly, in the present study, the TMCD, PMCD and IMCD increased in grade II tumours compared with grade I tumours, but decreased again in grade III tumours. Similar results have been seen in another study of CMTs counting overall MCD [18]. In dogs, no association has been found between TMCD or PMCD and grade, while low IMCD has been found to positively correlate with the grading [18, 27]. In agreement with published data, a significant association was only found between grade I and II carcinomas and PMCD. Furthermore, PMCD was not associated with any other parameter observed. As previously postulated, peritumoural MCs seem to have contradictory effects in BC, probably depending on other tumour microenvironmental factors. However, it seems they may have anti-cancer effect in CMTs because PMCD was not associated with a shorter DFS time in the present study or with OS in previous research [8].

In contrast, a high IMCD, TMCD and grade were found to be associated with a shorter DFS time in this study. However, only IMCD and grade remained as independent prognostic factors after multivariable Cox regression analysis. These findings corroborate previous findings showing a high association between the grade and shorter OS and DFS time in CMTs [3, 37, 38]. With regard to IMCD, low stromal mast cell density was found to be associated with shorter OS time due to metastasis in one previous study of MC density as a prognostic factor in CMTs [8]. The present study is in agreement with previous studies that only intratumoural (stromal), but not peritumoural or peripheral, MC count predicts poor prognosis of CMTs, but studies differ on the value of MC density. This contradiction is seen in BC research [10]. Some studies have found high MC density to predict poor survival [12, 39], while others have found that it shows a good prognosis [34, 40]. It seems that similar contradictions may be seen in CMTs. This difference could stem from the ability of MCs to secrete various mediators with opposing effects [41–43]. For example, MCs can promote carcinogenesis by secreting mediators such as VEGF or interleukin-8 that promote angiogenesis, and proteases that enhance metastasis [41]. Another factor that may influence the different behaviour of tumours with a different MC density could be the molecular phenotype of the neoplasm. A study of BC found a positive correlation between oestrogen receptors and MCs that could suggest a chemotactic activity for MCs by oestrogen [39]. Other studies have found a significantly different number of MCs between various molecular subtypes of BC [34, 44, 45]. In the present and previous studies of MCs in CMTs, the molecular subtypes of the tumours are unknown. Further studies are needed to investigate the association between MC density and the molecular phenotype of CMTs.

Furthermore, IMCD increased in large tumours, tumours with a high TIL count and tumours with lymph node metastasis at the time of surgery. With regards to nodal involvement, studies in BC found contrasting results with the association with IMCD, again showing the contradictory results of MC involvement in carcinogenesis [12, 34]. In the present study, average tumours (3–5 cm) had a lower count of intratumoural MCs than small (< 3 cm) tumours, but the density increased in large (> 5 cm) tumours. Similar results have been observed in invasive BC, however the difference was not significant [45]. In terms of TILs, activated MCs are known for their capacity to recruit immune cells to the tumour microenvironment by producing various chemokines and cytokines, which could explain the association between high IMCD density and high TILs. Similar results have been observed in metastatic renal cell carcinoma, but as the authors of that study noted, the exact chemokines and paired receptors are not known, thus any connection between these types of cells can only be hypothesised [46]. A high TIL count in BC is considered a good prognostic factor for DFS in HER2-enriched molecular and triple-negative subtypes, but not in the luminal molecular subtype [47]. The results in CMTs are scarce and contradict those of BC. In one study, CMTs with low TILs a had significantly higher OS than neoplasm with high TILs count [48], while in another study tumours with intratumoural low TILs count also had significantly higher OS, while peripheral TILs count did not have an association with OS [49]. The cause for the difference in BC and CMTs results could be the much bigger sample sizes in studies done with BC with a better analyzation of the results by meta-analysis. In CMTs just a few studies with relatively small sample sizes researching TILs as prognostic factor were done. In the present research, TIL count did not have an association with DFS. Relatively small sample sizes could be the cause why TIL or any of the other observed parameters except grade did not have an association with DFS.

TMCD, PMCD and IMCD were not associated with PI. No correlation between MCs count and PI was found in BC too [11]. In CMTs these parameters were not researched together. However, PI was associated with grade, mitotic index and presence of necrosis, showing the importance of this parameter in the assessment of tumour aggressiveness. These results are in agreement with a study in CMTs [5]. Albeit in our study PI was not associated with poor prognosis in disagreement with previously mentioned study. Several reasons could be behind the difference in the results. The previous study had 8 more samples of malignant CMTs compared to the follow-up in our study, but they did not include grade I carcinomas. Also, mean PI of grade III carcinomas in the mentioned study was higher by 9%, which can mean that tumours were more aggressive in the other study.

As MCs promote angiogenesis, MVD and EA were also counted. Angiogenesis is important for tumour growth and metastasis as the growing tumour requires more nutrients and oxygen, and necrosis develops in the parts deficient in blood supply [50]. This study found a positive significant correlation between MVD and EA, which is in agreement with a study of dogs with non-Hodgkin’s lymphoma [24]. No previous studies have investigated the association between these two parameters in CMTs. In agreement with a previous study, MVD correlated with TMCD, confirming the involvement of MCs in angiogenesis of CMTs [18]. Furthermore, in the present study MVD correlated with PMCD, but not with IMCD. This could be explained by the interesting feature of mast cells increasing around tumours just before the time when new capillaries start to grow towards the tumour [51]. Interestingly, no association was found between MVD and clinicopathological parameters, except where necrosis is present, whereas EA was associated with tumour grade, mitotic index, tubular formation and proliferation index. Similar associations between a higher MVD and the presence of necrosis have been found in BC and CMTs [21, 52]. As angiogenesis is important for tumour progression, it leads to increased growth of the tumour cells in particular areas, resulting in the formation of focal ischemia since the proliferation of tumour cells surpasses the formation of new blood vessels [52, 53]. In all cases, EA increased with the increase in parameters that show tumour aggressiveness. Similar results have been found in the above-mentioned study of canine non-Hodgkin’s lymphoma and in feline post-injection fibrosarcoma [24, 54]. In a study of CMTs, the median blood vessel area (BVA) had an association with OS time [19]. Furthermore, a significant association between grade and EA was found in BC [22].

A shorter DFS time was found to be associated with a high EA, but not with MVD in this study. Higher EA was found to be significantly associated with shorter DFS and OS times in BC, while the manually counted number of vessels was associated with just OS. Furthermore, in contradiction to the present study, EA has emerged as an independent prognostic factor [22]. In one study of CMTs, MVD had a predictive value for survival (DFS and OS) in benign tumours only, while in another it was associated with a shorter OS time [19], but an association with DFS time was not investigated [55]. High MVD is considered a sign of poor prognosis in BC, but there is still some discrepancy in the results of the various studies [56], and one meta-analysis concluded that while MVD can confidently be considered as a prognostic factor, its importance is debatable [57]. Therefore, MVD as a prognostic factor should be considered with caution in CMTs, especially when observing DFS time. In contrast, EA should be included as a parameter that can show tumour aggressiveness and could be a potential prognostic marker. It should be noted that the study population of the follow-up in this study was small, therefore further research into EA in CMTs would be of interest as only one piece of research has previously investigated this parameter in CMTs as prognostic [19].

## Conclusions

The findings expand the knowledge on tumour microenvironment and MCs in peritumoural and intratumoural zones. Intratumoural mast cells appear to play an important role in the carcinogenesis of canine mammary gland, while peritumoural mast cells does not seem to be implicated in it. IMCD and grade were found to be independent prognostic factors for disease-free survival. These results suggest that IMCD, especially with grade, could be used for a better prediction of progression of CMTs. However, these results differ from the only previous prognostic study on MC density of CMTs, showing that further research with bigger sample size is required into whether high or low intratumoural mast cell density is a predictor of poor prognosis and which other tumour characteristics might influence this divergence. Furthermore, this study suggests that EA could be an additional factor that could add value in determining tumour aggressiveness and progression.

## Data Availability

The datasets used and/or analysed during the current study are available from the corresponding author upon reasonable request.

## Abbreviations

BC: Breast cancer
CMC: Canine mammary carcinoma
CMT: Canine mammary tumour
DFS: Disease-free survival
EA: Endothelial area
EPOR: Erythropoietin receptor
H&E: Hematoxylin–eosin
HPF: High power field
IMCD: Intratumoural mast cell density
IQR: Interquartile range
MC: Mast cell
MVD: Microvascular density
OS: Overall survival
PI: Proliferation index
PMCD: Peritumoural mast cell density
SD: Standard deviation
TIL: Tumour-infiltrating lymphocytes
TMCD: Total mast cell density
VEGF: Vascular endothelial growth factor
